# On Scalds

**Published:** 1807-07

**Authors:** 


### To Dr. BATTY.

Dear Sir,

If I had pursued Physic as a profession, I think I should have become an excellent empyric, although I confess my impatience to get at the end, might have occasionally induced me to disregard the progress, and perhaps made me a bad practitioner. You know very well how often I have cut short your arguments by my impertinent interruption of, "Will it cure it"? Will this be the result? Is this a matter of fact, or only an opinion? S'death! it seems a scandal to the profession, that there can be two opinions on a point of practice at this time of the day, and I'm almost inclined to say, "Throw Physic to the Dogs, I'll have no more on't."—But as my laconic method of proceeding may not be quite so conformable to some high flown Theorists, who occasionally sport their technical terms and far-fetched epithets in the pages of your excellent Journal, I beg to relate a case that most *providentially* occurred in my family, and gave me an opportunity of putting two opposite modes of practice to the test; a thing I delight in. The result I submit to any of your long-winded correspondents, who choose to become the knights in the contest, not doubting but I shall be extremely edified with their lucubrations, if I am not convinced.

On Sunday evening, the 5th of April, I was suddenly roused from as sound a nap in my *chair*,* as I ever got at church in the morning, by the footman telling me my *maid's*

---

* Dr. Batty requests his lively correspondent T. Y. to accept his thanks for this communication on Scalds, and also for his former one on Psoriasis Diffusa, which afforded a very satisfactory proof of the powerful effects of Arsenic.

*maid's* child had scalded both his legs and thighs in the most dreadful manner, by falling from a chair into a pan full of hot water. I had presence of mind to seize a bottle of turpentine the painters had left on the stairs, in my way down to the kitchen; and while I was anointing the thigh and leg with a feather, I ordered the other leg and thigh to be immersed in a jar of cold water. I need not mention the screams, fright, &c. on the occasion. I suppose they were as decently uttered, as those that take place under similar circumstances. In the midst of all the poor creature's sufferings, I felt delighted at the thoughts of bringing Dr. Kinglake and Dr. Kentish to such close quarters.—The case seemed a fair one, both limbs appearing equally bad; the blisters were very large, the penis and scrotum also suffered; and as I wished to divide the credit as equally as I could between the combatants, I applied turpentine to the scrotum, and water to the penis.

In about ten minutes the child was composed. He shivered, and when I was tired with anointing, he begged his mother to use it; he fell asleep with his leg in the jar, and as the water got warm, I emptied it with a small syphon, and had cold water poured in, that the limb might not be disturbed. The process continued all night.

(Monday.) The vesications of the watered limb appear less tense, and the small interstices not so vivid as on the other limb; he says he has not had much pain. Medicine was poured down to procure a motion, and while operating, the limb was obliged to be taken out of the jar; but in order to give it as fair a chance as possible, linen rags were wetted and applied to those parts where the streams from a watering pot could not touch; the leg was afterwards replaced. The other limb and scrotum were turpentined all day; in the evening the boy eat a little bread and milk. The footman and another servant attended him all the preceding night, and followed my directions to a tittle, but he was very restless and feverish, and got no sleep; however, the limbs were not worse; he passed this day tolerably well, but had a smart rigor when the water was changed in the evening, and became excessively hot and feverish in the night, with pulse above 130; great thirst and restlessness..—The penis and the watered leg this morning, Tuesday

Arsenic. At the same time, he begs leave to observe, that cases in Physic and Surgery, would be quite as acceptable, if drawn up with the seriousness the subject demands.

(Tuesday,) look not so kindly as the other, and make me doubt either its salutary effect, or the possibility of carrying on a double plan of cure in the same patient. As I wished to divest my mind of all partiality for any mode, while there seemed no danger to life from the application of both, I felt anxious for a continuance of the double plan I had directed; at the same time, I was extremely sensible that an amateur had no right and title to sport with the lives of his Majesty's liege subjects; and this consideration damped the fun I hugged myself on enjoying in the battle between the learned Doctors, and the benefit I had reason to believe society would derive from the experiment. However, I thought if things did not mend before night, I would send for further assistance, and till that time, I should go on with my separate applications.

At night, the maid told me the child could not make water, and that his small affair was as black as her *bonnet*. I took horse and rode for my Æsculapius, who laughed very heartily at the impossibility of my experiment deciding the question, while the same limbs were under the influence of one constitution. This thought, I own, did not occur to me, and at the same time, I thought not of the importance he attributed to it; he agreed, that in slight accidents, as a cut or bruise on each hand, it might not interfere with two opposite plans; but in this case, the injury was so extensive, the whole system was engaged in the danger. The urine was drawn off, and after a careful inspection of the limbs, he determined on abandoning the cold water, and pursuing the turpentine; for he assured me the penis and watered limb would mortify, without a different application. Turpentine was now applied to all the injured parts, and it has been persisted in with the addition of yellow bazilicon; the boy is now mending very fast with the loss of his prepuce, for certainly no Jewish priest could ever have circumcised him more neatly than this accident.

The most vexatious part of the affair is, that the dispute is not settled; and I shall not feel content, till some practitioner meets with two scalded patients, and gives both plans a fair trial; and what is almost as bad, the hopes of edification I expressed in the beginning of this letter (which was begun a few days after the injury) cannot be realized, because the data are now done away with. If I say, (which is the fact) that the limb first turpentined, is much forwarder than the other; Dr. Kinglake can very readily reply, that the other had not a fair chance, as the

 water water was not repeated. On the whole, I should be doubtful of the utility of publishing such a case, if it was not for the wish I have, that surgeons would endeavour fairly to try the experiment, and not bring us their cargoes of proofs of the truth of which there may be no doubt. I am inclined to believe, the human constitution is a much more good natured complying machine than is generally thought of.—Brown paper and vinegar seems to cure a bruised forehead, as readily as a solution of sal. ammoniac; and it is an even bet, that port wine, or scraped potatoes, would do it as well. I am told, the late Mr. Hunter cured gonorrhœas with bread pills as speedily as with calomel; and I certainly cured one without any application or internal medicine, quite as quickly as when I had the best advice, and used injections and medicine in abundance. I have no wish to carry this idea beyond its proper limits; I told you before I was an amateur, and shall feel obliged to any of your numerous correspondents for their sentiments, after having made fair comparative trials of the superior efficacy of either remedy in burns or scalds.

